# The Malaysian Food Barometer 2 dataset: bridging socio-anthropology and nutrition through extended 24-h recall

**DOI:** 10.3389/fnut.2025.1713959

**Published:** 2026-01-02

**Authors:** Elise Mognard, Cyrille Laporte, Laurence Tibère, Norimah A. Karim, Yasmine Alem, Khairul Hasnan Amali, Khadidja Chekima, Helda Khusun, Judhiastuty Februhartanty, Adam Drewnowski, Anne Dupuy, Amandine Rochedy, Kremlasen Naidoo, Jacqui Kong, Salini Devi Rajendran, Nurliyana Jekria, Theresia Pratiwi Elingsetyo Sanubari, Jan Li Yuen, Neethiahnanthan Ari Ragavan, Mohd Noor Ismail, Jean-Pierre Poulain

**Affiliations:** 1 Faculty of Social Sciences and Leisure Management, Taylor's University, Subang Jaya, Malaysia; 2 Chair “Food Studies: Food, Cultures & Health”, Taylor's University, Subang Jaya, Malaysia; 3 Center for Asian Modernisation (CAM), Taylor's University, Subang Jaya, Malaysia; 4 Centre d'Études et de Recherche: Travail, Organisation, Pouvoir (CERTOP) UMR CNRS 5044, Université de Toulouse, Toulouse, France; 5 Marches Organisations Institutions et Stratégies d'Acteurs UMR MOISA, Montpellier, France; 6 Institut de Recherche pour le Développement (IRD), Sainte Clotilde - La Réunion, France; 7 Faculty of Health Sciences, Centre for Community Health Studies (ReaCH), Universiti Kebangsaan Malaysia, Bandar Baru Bangi, Malaysia; 8 Centre for Nutrition Epidemiology Research, Institute for Public Health, Ministry of Health, Shah Alam, Malaysia; 9 Faculty of Food Science and Nutrition, Universiti Malaysia Sabah, Kota Kinabalu, Malaysia; 10 Southeast Asian Ministers of Education Organization Regional Center for Food and Nutrition (RECFON) – Pusat Kajian Gizi Regional Universitas Indonesia, Jakarta, Indonesia; 11 Faculty of Health Sciences, University of Muhammadiyah Prof. Dr. HAMKA, Jakarta, Indonesia; 12 Faculty of Medicine, Nutrition Department, Universitas Indonesia, Jakarta, Indonesia; 13 Center for Public Health Nutrition and Department of Epidemiology, University of Washington, Seattle, WA, United States; 14 Program Studi Gizi, Fakultas Ilmu Kesehatan, Universitas Kristen Satya Wacana, Salatiga, Indonesia

**Keywords:** open science, sociology of food, anthropology of food, nutritional epidemiology, health, food practices, transition, compressed modernization

## 1 Background

The Malaysian Food Barometer (MFB) was established to provide integrated, nationally representative datasets combining socio-anthropological and nutritional perspectives on changes in habits in Malaysian multicultural society. It aims to understand how modernization shapes the food lifestyles and different socio-cultural determinants of decision-making processes, while also analyzing the implications of these changes for public health consequences, particularly regarding non-communicable diseases, as well as for the food service sector (1, 2). It was established, in the frame of the chair “Food, Cultures and Health” jointly set up by Taylor's University (Malaysia) and Toulouse University (France) and lead by Jean-Pierre Poulain. The first wave (MFB1) demonstrated the value of linking food day's organizations, meal patterns, social context, and food practices to understand transformations of food habits and their consequences in terms of health including obesity (2, 3). Following the open science philosophy, all the works of Asian Food Barometer initiative dataset are reported (4, 5) and the scientific reports are openly available within the series “Food Trends in Southeast Asia” (6, 7).

The second wave (MFB2) builds on the conceptual and methodological foundations established in MFB1, incorporating advances in both the sociology and social anthropology of food as well as in nutritional epidemiology. The major innovation introduced in this second edition is the refinement of the 24-h dietary recall methodology, which now enables the simultaneous collection of detailed nutrient intake data, social-contextual dimensions of eating practices, and four distinct categorizations of protein sources, including one that addresses whether killing for food is required. This adaptation reinforces the bridge between social science approaches and nutritional analysis, thereby generating a dataset capable of supporting interdisciplinary research on food transitions in Malaysia.

This article provides a systematic overview of the MFB2 dataset. We first describe the study design and sampling procedures, followed by the survey instrument and the extended 24-h recall methodology. We then present the data collection and processing protocols, detail the dataset structure and derived variables, and conclude with illustrations from published analyses together with perspectives for future research applications.

## 2 Methods

### 2.1 Study design and sampling

A cross-sectional survey was conducted between March and July 2018 on a nationally representative sample of Malaysian residents aged ≥18 years (*N* = 1,604). Data collection was scheduled to avoid major religious and festive periods in Malaysia, ensuring that the recorded food practices reflected habitual, non-festive conditions.

The sampling frame was stratified by region (Peninsular Malaysia, Sabah, Sarawak), degree of urbanization (urban, suburban, rural), gender, age group, and ethnicity, following quotas aligned to the national census. Respondents were randomly selected within strata. Details on sample calculation are provided in Table 1.

**Table 1:** Summary of MFB2 sampling design.

**Strata**	**Categories**	**Quota basis**
Region	Peninsular Malaysia, Sabah, Sarawak	Census proportion
Urbanization	Urban, suburban, rural	Census definitions
Gender	Male, Female	National distribution
Age groups	18–25, 26–35, 36–45, 46+	National distribution
Ethnicity	Malay, Chinese, Indian, Non-Malay Bumiputra	National distribution

### 2.2 Overview of the research instrument

The MFB2 survey combined:

- Socio-demographic & Economic variables: include gender, age, ethnicity, religion, marital status, household size, number of children, education level, occupation, income per capita, and income change over the past 5 years.
- Social representations variables: Respondents identified components of a “proper” meal, ranked perceived food risks, and reported social representations about health-promoting and health-harming foods.
- Practices variables: Covered eating out (week prior, day prior), cooking practices, commensality, activities during meals, and structure of eating events. These practices are mostly collected with the extended 24-h dietary recall, incorporating nutritional and socio-contextual details and further elaborated below.

While structured questionnaire items ensure standardization and comparability, several components of the instrument were intentionally designed as open-ended to preserve cultural nuance. In particular, within the 24-h recall, the *name of the intake* is recorded verbatim and not pre-coded. This allows respondents to describe eating events in their own terms, maximizing cultural expressiveness and avoiding the imposition of a normative structure on the food day. However, as with all structured survey instruments, some fine-grained cultural subtleties may not be fully captured. The combination of structured items and targeted open-ended components represents a compromise between standardization, necessary for comparisons, and the preservation of cultural specificity.

### 2.3 Extended 24-h recall: bridging methods

#### 2.3.1 Conceptual foundations: *food social space* and *food day structures*

The concept of the *Food Social Space*, developed by Jean-Pierre Poulain as an extension of Georges Condominas' notion of *Social Space* (8–10), conceptualizes the interactions between food cultures and nature—understood both in biological/physiological terms and in relation to the environment. It provides a framework for describing the social dimensions of food cultures, including forms of consumption (meals and between-meal intakes), modes of eating (table manners), and commensality together with the rules that govern it (the act of eating with others).

Within debates on the modernization of food habits, the *Food Social Space* has offered an empirical foundation for the systematic comparison of food-day structures and eating events in both social norms and practices in France (11, 12); for the study of dietary transformations and the rise of obesity in rapidly modernizing contexts such as French Polynesia (13) and La Reunion (14–16); and for analyzing food socialization (17–19).

By clearly distinguishing the elicitation of social norms (“proper meal”) from the reconstruction of actual intakes, the two-step approach reduces both desirability bias (responding according to expected norms) and conformity bias (responding according to perceived group norms). The explicit instruction that the second step concerns “what actually happened yesterday” further facilitates recall accuracy by releasing respondents from normative pressure.

#### 2.3.2 Research context in Malaysia

Knowledge of dietary habits in Malaysia is derived primarily from nutritional and ethnological research. Quantitative nutritional research has mainly concentrated on the composition of food intake and its association with body weight status (20–28). Qualitative ethnological studies, in turn, spans from concentration on traditional systems of beliefs and eating practices linked to key life events (29) to the impacts of globalization, urbanization, and tourism on local foodways (30).

One of the methodological innovations of the Food Barometers has been to translate these ethnographic observation tools into quantitative surveys aiming at facilitating dialogs with researchers from nutritional epidemiology, economics and societal stakeholders. Researchers have adapted methodologies, whether through participant observation of eating events, or indirect observation of sociotechnical catering arrangements of eating practices and catering settings (including mobile street vendors) to serve as the starting point for the survey questionnaire design.

#### 2.3.3 Transition models and modernization of food habits

Transition models (31), inspired by the demographic transition framework, describe the structural transformations of societies, with phases marked by a lag between prevailing values and norms on the one hand, and material living conditions on the other (32). In the study of food modernization and its associated transitions, longitudinal analyses make it possible to examine processes such as the individualization of eating events as a key consequence of these changes. Individualization (or de-socialization) of eating can be observed at several levels within the food day: first, through the modalities of synchronization of eating events; second, through the extent of their socialization; and third, at the level of dishes themselves, as illustrated by socio-historical approaches to gastronomy and table manners.

The 24-h recall method is widely used in nutrition and public health as a standardized approach to documenting food intake. Within the Malaysian Food Barometer, this tool was adapted to address its limitations by distinguishing between normative descriptions of “proper meals” and reconstructions of actual intake. By incorporating questions on the social context, synchronization, and socialization of eating events, the method captures not only quantitative data but also the socio-cultural organization of the *food day* (33), understood as being organized around meals considered fundamental elements of the social organization of eating (34, 35). *Food day* structures vary across societies and, within a single society, across social groups and contexts (13, 36), and evolve differently depending on their permeability to nutritionalization and changing food environments (37, 38) which invites us to adopt varied context scales.

#### 2.3.4 Implementation in MFB2

In MFB2, the 24-h recall was redesigned to collect both nutritional quantities and social-contextual variables for every eating event. Each participant contributed one 24-h recall covering the previous day. This was achieved through:

- Time-sequenced intake reconstruction—from waking to sleeping, capturing all eating or drinking events (except water). The formalization of the event is then recoded based on the name of the intake declared by the respondent, and where not sufficient, its time-implantation and sequence within the food day and social-context variables.
- Social-context variables—location, origin of food, co-presence of others, activities during intake.
- Dishes eaten—respondents are prompted on their individual and collective dishes consumption.
- Quantitative detail—portion sizes assessed with the *Album Makanan Malaysia* and coded into the *NutritionistPro database* (2017 Malaysian Food Composition Table, 1,892 foods, 29 mandatory nutrients).
- Protein sources categorization—each food is assigned to protein source categories, enabling multi-disciplinary theoretical framings (e.g., plant vs. animal; fisheries focus; animal requiring vs. not requiring killing).

This dual capture transforms the 24-h recall into a “bridged” tool that supports both nutrient-level analyses (energy, macronutrients, micronutrients) and sociological studies (meal sociality, socio-cultural norms, urbanization effects).

In practice, the interviewer facilitates recall of all activities from waking to sleeping. Each time an eating or drinking event (excluding water) is identified, a series of questions on its social organization and content is asked. The number and naming of eating events vary across respondents, enabling the reconstruction of food day structures during analysis. A first qualitative step categorizes eating events by name and degree of institutionalization, following Herpin's criteria (34)—concentration, timing, synchronization, location, and ritualization—allowing eating events to be positioned along a continuum from highly institutionalized (main meals such as lunch or dinner) to weakly institutionalized (snacks). Small meals such as teatime or supper occupy an intermediate position. This can be complex, as names appear in multiple languages (notably English and Malay). Repetition of a meal label (e.g., two breakfasts) within a single day raises questions about its degree of institutionalization. A second analytical step examines the temporal distribution of the proportion of the (sub)population consuming each type of event, identifying dominant structures and their variation across socio-cultural groups. At the national level, Malaysian food days are generally structured around two or three main meals, with small meals (teatime) having relatively institutionalized timing. Each eating event's content is further distinguished into shared and individual components. This distinction both assists recall and serves an analytical function: practices are categorized as “individual” if only individual components are mentioned, or “collective” if collective components, or a combination of collective and individual components, are reported.

### 2.4 Data collection and procedures

Enumerators held degrees in nutrition or social sciences and received dedicated training in both components of the research instrument. Training was conducted across three sessions of 15 participants each on 26 March and 21 June 2018 and included: standardization of interview procedures, practice of the semi-directive administration of the 24-h recall and troubleshooting of likely field challenges.

To mitigate participant fatigue during interviews, the questionnaire alternated between closed-ended and open-ended questions to vary cognitive load. Instructional prompts were inserted at several transition points to break the pace and support re-engagement. Finally, the 24-h recall was administered face-to-face using a semi-directive interview approach, allowing interviewers to adapt the rhythm of questioning, provide clarification when needed, and monitor signs of fatigue. These combined strategies helped maintain respondent attention throughout the interview.

Data entry used double-check protocols. All foods and recipes reported in the 24-h recalls were encoded and analyzed for energy, macronutrients, and micronutrients using NutritionistPro version 8.1, which incorporates the Malaysian Food Composition Table (MyFCD, updated 2017). When data were unavailable in the MyFCD, values were supplemented from the United States Department of Agriculture (USDA) National Nutrient Database (39). Implausible energy intakes (< 500 or >4,000 kcal/day) were flagged and excluded from nutrient analyses. More elaborate methods, such as the Goldberg cut-off incorporating basal metabolic rate, were not applied here. Users should therefore remain cautious about the possibility of under-reporting near this threshold. As with all self-reported dietary data, some degree of general underestimation of intake may persist, particularly among population subgroups typically prone to underreporting. Moreover, no adjustment for physical activity level or basal metabolic rate was performed to evaluate the plausibility of reported energy intakes, which may further limit the assessment of reporting accuracy. All statistical analyses were performed using Statistical Package for the Social Sciences (SPSS) version 26 (IMB, New York, NY, USA).

A quality monitoring session with six enumerators was additionally conducted to ensure inter-interviewer and coding consistency. This process helped strengthen the reliability of both socio-anthropological and nutritional components of the survey.

## 3 Data records

In sum, the MFB2 dataset comprises socio-demographic & economic, geographic & urbanization, social representations, food practices (name of the intake, time, location, preparation, commensality, activities, individual and collective dishes consumed), and health status (BMI as a proxy). Users analyzing the dataset should distinguish between variables capturing normative representations (e.g., “proper meal” components) and variables capturing observed intake from the 24-h recall, as they serve analytically distinct purposes.

## 4 Already published results, work perspectives, and discussion

Some results illustrative of the cross-disciplinary research on the compressed modernization of the Malaysian and South-East Asian food habits have been produced and published around the specific problematics of the protein transition, as part of the Southeast Asian Consortium for Research in Protein Transition (SCRiPT) project (40–43) and of the breakfast recommendations, as part of the International Breakfast Research Initiative (IBRI) (44–47). But the interest of the MFB database is far from exhausted since MFB2 enables analysis of social representations such as healthy foods, national dietary surveillance with socio-cultural dimensions, analysis of food and protein transition by multiple disciplines and theoretical framings, cross-national comparison of the impacts of modernization and change dynamics within Food Barometers and longitudinal comparison with MFB1 to assess changes in food related social representations and practices 2013–2018. This interdisciplinary integration enables novel analyses that relate cultural *food day* patterns, commensality, and synchronization of eating events to nutrient intake profiles. Such linkages would not be visible in conventional nutrition surveys, demonstrating the analytical value added by embedding socio-anthropological concepts within dietary recall.

Several methodological limitations should be acknowledged. First, the 24-h recall is inherently subject to recall errors and under- or over-reporting, even though the two-step design helps mitigate desirability and conformity biases. Small eating events, condiments, and beverages are especially prone to omission in declaration, a limitation that users should consider when analyzing specific food groups. Second, each participant contributed only a single recall day, which limits the assessment of habitual intake and does not allow examination of weekday–weekend variation; because data collection occurred from Tuesday to Saturday, only intakes from Monday to Friday are represented. Third, the survey includes only adults aged ≥18 years, thereby excluding children and adolescents and limiting the range of life stages captured. Moreover, the dataset does not include physical activity information or clinical health indicators beyond BMI. As a result, while the data support analyses linking dietary practices to socio-demographic and socio-cultural factors, they do not permit direct evaluation of diet–disease relationships or full clinical assessment of nutritional status.

## 5 Conclusion

The Malaysian Food Barometer 2 (MFB2) extends the scope of national dietary surveys by explicitly bridging sociology, anthropology, and nutritional epidemiology. Its extended 24-h recall methodology was designed to support parallel analyses across disciplines, providing not only nutrient-level data but also detailed information on preparation, commensality, and the social organization of eating events. By incorporating multiple categorizations of protein sources, the dataset enables nuanced investigations into the protein transition, including distinctions between plant and animal proteins and between animal foods requiring or not requiring killing (animal products such as eggs and poultry for example). Compatibility with MFB1 ensures that researchers can conduct longitudinal analyses, assessing how food practices and representations have evolved over a 5-year period in the context of Malaysia's compressed modernization. Through this integration of nutritional quantities with contextualized social data, MFB2 offers a unique open dataset to advance interdisciplinary research on food transitions and their health, social, political and cultural implications in Southeast Asia.

## Data Availability

The datasets presented in this study can be found in online repositories. The names of the repository/repositories and accession number(s) can be found in the article/Supplementary material.
