# Supplementary material for: The Malaysian Food Barometer 2 dataset: bridging socio-anthropology and nutrition through extended 24-h recall
Source: Front Nutr. 2026 Jan 2;12:1713959. doi: 10.3389/fnut.2025.1713959 (PMC12807946; doi:10.3389/fnut.2025.1713959)
Supplement: Supplementary file 3 [file Data_Sheet_2.docx]

| Respondent Name |  | | |
| --- | --- | --- | --- |
| Address |  | | |
|  |  | | |
| City |  | Postcode |  |
| Contact no | (O) | (H) | (HP) |
| Email |  | | |
| Date |  | | |
| Time Start |  | Time End |  |
| Interviewer |  | | |

| Z1 North | Code |  | Z2 Central | Code |  | Z3 South | Code |
| --- | --- | --- | --- | --- | --- | --- | --- |
| ~~Perlis~~ | ~~1~~ |  | Perak | 4 |  | N. Sembilan | 7 |
| Kedah | 2 |  | Kuala Lumpur | 5 |  | Melaka | 8 |
| Penang | 3 |  | Selangor | 6 |  | Johor | 9 |

| Z4 east Coast | Code |  | Z5 East Malaysia | Code |
| --- | --- | --- | --- | --- |
| Pahang | 10 |  | Sabah | 13 |
| Terengganu | 11 |  | Sarawak | 14 |
| Kelantan | 12 |  | ~~Labuan~~ | ~~15~~ |

| Q2.Gender | Code |  | Q3. Living area | Code |  | Q6 | Code |
| --- | --- | --- | --- | --- | --- | --- | --- |
| Male | 1 |  | Urban | 1 |  | Non – Malay Bumiputra | 1 |
| Female | 2 |  | Suburban | 2 |  | Malay | 2 |
|  |  |  | Rural | 3 |  | Indian | 3 |
|  |  |  |  |  |  | Chinese | 4 |
|  |  |  |  |  |  | Other | 5 |

| **Introduction** |
| --- |

| Good morning / afternoon / evening, my name is ____________. We are conducting a survey on behalf of Taylor’s University in line with the research initiative Of Ministry of Higher Education Malaysia.  The objective is to study Malaysian food habits and their changes. (More specifically, this project focuses on the cultural and economic aspects of the “protein transition”.) （Read only if needed）  This research focuses on people of aged 18 and above. Full anonymity and confidentiality are guaranteed.This questionnaire will take approximately 45 minutes.  早安 ／ 午安 ／ 下午 好，我的名字是 XXX .  我们正为大马教育部进行在进行一项关于大马人饮食习惯与转换的调查访问。(它主要是针对文化上与经济上的“蛋白质摄取”)  我们保证不会泄漏您的资料。我们希望您可以参与这份大约45分钟的问卷回答。  *Selamat pagi / tengah hari / petang, nama saya _______. Kami sedang menjalankan kajian bagi phak Taylor’s University sejajar dengan inisiatif penyelidikan Kementerian Pendidikan Tinggi Malaysia.  Objektifnya adalah untuk mengkaji tabiat makan penduduk Malaysia dan pertukarannya. (Lebih spesifik, projek ini memberi tumpuan kepada aspek kebudayaan dan ekonomi terhadap transisi protein.)(Bacakan jika perlu)*  *Kajian ini ditujukan untuk golongan respondent yang berumur 18 tahun dan ke atas.  Maklumat yang diberikan ini akan hanya digunakan bagi tujuan kajian sahaja dan maklumat responden adalah sulit.*  *Soal selidik ini akan mengambil masa lebih kurang 45 minit.* |
| --- |

| **Section A: Filter Questions** |
| --- |

| Q1 | Malaysian citizen  马来西亚公民  *Warganegara Malaysia* | SA | Route |
| --- | --- | --- | --- |
|  | Yes是Ya | 1 | Continue |
|  | No不是Bukan | 2 | Terminate |

| Q2 | Gender性别Jantina (Ask only if needed) | SA | Route |
| --- | --- | --- | --- |
|  | Male男Lelaki | 1 | Check Quota |
|  | Female女Perempuan | 2 |  |

| Q3 | Living area  生活地区  *Tempat tinggal* | SA | Route |
| --- | --- | --- | --- |
|  | Urban  城市  Bandar | 1 | Check Quota |
|  | Sub Urban  镇  *Pekan* | 2 |  |
|  | Rural  乡村  Luar bandar | 3 |  |

| Q4 | Date of Birth and Age  生日日期与岁数  *Tarikh Lahir and Umur* | Code | Route |
| --- | --- | --- | --- |
|  | Date of Birth:  Tarikh Lahir |  | Check Quota |
|  | Age:  Umur |  |  |

| Q5 | Ethnic group or race种族Kumpulan Etnik atau Bangsa (do not read first) | SA | Route |
| --- | --- | --- | --- |
|  | Non – Malay Bumiputra  非巫族土著  Bumiputera bukan Melayu | 1 | Check Quota |
|  | Malay  巫族  *Melayu* | 2 |  |
|  | Indian  印族  *India* | 3 |  |
|  | Chinese  华族  *Cina* | 4 |  |
|  | Others  其他  *Lain-lain* | 5 |  |

| **Section B : Norms on Food** |
| --- |

| Q6 | Generally speaking, how many main meals are you consuming per day?  您通常一天内吃多少份正餐呢？  *Secara umum, berapa hidangan utama yang anda makan setiap hari?* | SA | Route |
| --- | --- | --- | --- |
|  | 1 meal per day / 1天1餐/ *1 hidangan setiap hari* | 1 | Continue |
|  | 2 meals per day / 1天2餐 / *2 hidangan setiap hari* | 2 |  |
|  | 3 meals per day / 1天3餐 / *3 hidangan setiap hari* | 3 |  |
|  | 4 meals day / 1天4餐 / *4 hidangan setiap hari* | 4 |  |
|  | More than 4 meals / 超过4餐/ *Lebih daripada 4 hidangan setiap hari* | 5 |  |

| Q7 | Besides those main meals, how often do you have food intakes in between meals (tea break, snacks, etc.)?  除了这些正餐以外，您是否还有经常进食呢？（茶点，零食等等）  *Selain daripada hidangan utama tersebut, apakah kekerapan pengambilan makanan sampingan anda (Minum Petang, snek, etc)* | SA | Route |
| --- | --- | --- | --- |
|  | Never / 没有 / *Tak Pernah* | 1 | Continue |
|  | Once to twice a day / 1天1或2次 / *Satu hingga dua kali sehari* | 2 |  |
|  | Three to four times a day / 1天3至4次 / *Tiga hingga empat kali sehari* | 3 |  |
|  | Five to six times a day / 1天5至6次 / *Lima hingga enam kali sehar*i | 4 |  |
|  | More than six times a day / 1天超过6次  */ Lebih daripada enam kali sehari* | 5 |  |

| Q8 | The following question is about your usual meal organization. Could you please explain what the following meals usually consist of? Show card.  接下来的问题是有关于您用的餐单。我将会问一些有关于您的早餐，午餐，晚餐，宵夜和茶点的餐单。  *Soalan yang berikut adalah berkaitan organisasi hidangan yang biasa anda makan. Boleh anda jelaskan setiap hidangan makanan yang anda makan (sila rujuk kad)* | | | | | | |
| --- | --- | --- | --- | --- | --- | --- | --- |
|  | A proper breakfast  全早餐  *Sarapan pagi yang betul* | A proper brunch  (Breakfast and Lunch taken as one meal)  全早午餐  Makan (sarapan pagi dan makan tengah hari diambil sebagai satu sarapan)  yang betul | A proper lunch  全午餐  *Makan tengah hari yang betul* | A proper dinner  全晚餐  *Makan malam yang betul* | A proper supper  全宵夜  *Makan*  *lewat malam yang betul* | A proper snack  茶点  *Snek* | A proper tea time  下午茶  *Watuk minum* |
|  |  |  |  |  |  |  |  |

| **Section C : Food intakes of the last 24 hours** |
| --- |

| Q9 | Before we proceed to recall your food consumption for yesterday, we would like you to a recall your food consumption of the previous one week, before yesterday. It is not uncommon to forget what you have eaten for the past week. However I would like you to try to remember how many meals you had at your home for the last seven days.  在我们还没有继续有关于您昨天所吃过的食物，现在让我们想想您前几天所享用过的食物。  虽然要想回数天前吃过的食物并不简单。但是我希望您可以尽量回想您过去前7天里所吃过所有餐点的次数。  *Sebelum kita teruskan dengan soalan pengambilan makanan anda semalam, kami ingin anda cuba ingat kembali pengambilan makanan anda seminggu sebelumnya. Bukanlah suatu perkara yang luar biasa sekiranya anda lupa, walau bagaimanapun, saya ingin anda cuba ingat seberapa banyak boleh, berapa hidangan anda makan di rumah anda untuk tujuh hari yang lalu* | SA | Route |
| --- | --- | --- | --- |
|  | I eat xx times at home last week  我一个星期前在家里总共吃了xx 餐  *Saya makan xx kali di rumah pada minggu lepas* |  | Continue |
|  | I eat xx times outside last week  我一个星期前在外头总共吃了xx 餐  *Saya makan xx kali di luar pada minggu lepas* |  |  |

[IF IT CAN FACILITATE THE ANSWER TELL “IF *IT’S EASER FOR YOU TO REMEMBER HOW MANY MEALS YOU HAD OUTSIDE YOU CAN TELL ME”.*

Probe:
More or less out of 21 meals (morning, noon, evening * 7 days)
Double check that the participant mentions the place of consumption (not of preparation of the food)*]*

[如果这能够帮助您回答这个问题，那“如果您是比较容易记得您曾经在外用餐的次数，那就告诉我在外用餐的次数吧

探测：

大约是21餐（上午，中午，晚上* 7天）或多或少

仔细检查参与者是否提及消费地点（而不是准备食物）]

JIKA BOLEH MENGINCILIKAN JAWAB TELAH "JIKA JUGA UNTUK ANDA UNTUK TELAH TELAH BAGAIMANA BANYAK MAKANAN ANDA TELAH DIBERIKAN ANDA BOLEH TELAH AKU".

Siasat:

Lebih kurang daripada 21 kali makan (pagi, tengah hari, petang * 7 hari)

Semak semula bahawa peserta menyebut tempat penggunaan (bukan penyediaan makanan)]

| If less than 21 meals, please double check that none of the meals has been left out. If not, ask the reason why less than 21 meals.  如果少于21餐，请仔细检查是否遗漏了任何餐点。如果没有，请询​​问为什么少于21餐*。*  *Jika kurang dari 21 kali makan, sila semak semula bahawa tiada makanan yang telah ditinggalkan. Jika tidak, tanyakan sebab mengapa kurang daripada 21 kali makan.* |
| --- |

| Q10 | Now I would like you to remember what and how you have been eating and drinking for the whole day yesterday from the time you wake up to the time you went to sleep.  It doesn’t matter if it wasn’t what you usually eat. It is important to me to understand the context in which you took your food. For this purpose, I will ask you several questions that can help you to remember your different food and drink intakes.  现在我希望您可以想想您在昨天所吃喝过的食物和饮料。无论那是不是您平时所享用的食物或饮料。  我非常希望可以详细了解您用餐时的餐单。  所以我将会询问一些可以帮助您回忆有关您所享用过的食物餐单的问题。  *Sekarang saya ingin anda ingat apa dan bagaimana anda makan dan minum sepanjang hari semalam.*  *Ia tidak mengapa jika ia bukan yang apa anda biasa makan.*  *Ia adalah penting bagi saya untuk memahami dan menganalisis persekitaran pengambilan makanan anda.*  *Untuk tujuan ini, saya akan tanya anda beberapa soalan yang boleh membantu anda mengingati pengambilan makanan dan minuman yang berbeza* |
| --- | --- |

| 1 Your first food or drink intake yesterday / *Pengambila nmakanan atau minuman pertmana anda semalam* / 您昨天的第一份食物或饮料 | | | | | | | |
| --- | --- | --- | --- | --- | --- | --- | --- |
| Where did you have your first food or drink intake yesterday? *Dimanakah anda mengambil makanan pertama atau minuman pertama semalam?*  你昨天在哪里吃了第一份食物或饮料？ | | Where did the food come from?  *Disediakan oleh siapa?*  是谁准备的食物？ | What is the name of this intake?  *Nama* Pengambilan makanan  您如何定义这一餐呢 | | | Could you describe the social context on this intake?  *Bolehkah anda menerangkan situasi anda semasa pengambilan ini?*  请说说您用餐时的情况 | |
| 1. At home  *Di Rumah*  *在家里* | 1. Your place *Tempat anda*   我家 | 1. Cooked by you  *Dimasak oleh saya*  我自己准备的 | At what time? *Pukul berapa?* 在什么时候 | | | 1. Alone  *Sendiri*  自己 | |
|  |  |  | What was the duration of this intake?  Berapa panjang masanya?  您用了多少时间 | | |  |  |
|  | 1. Friend’s place or relative’s house *Tempat Kawan atau saudaramara*   朋友或亲戚家 | 2. Cooked by friend / family  *Dimasak oleh kawan / keluarga*  朋友或家人准备的 | Could you describe the content(s) of your meal, food or drink intake?  *Bolehkah anda menerangkan kandungan makanan, atau minuman anda?*  请告诉我用餐的内容? | | | 2. With someone *  *Dengan seseorang **  与人共餐 | |
|  |  | 3. Delivery & brought from outside*  *Penghantaran & Bungkus/ Dibawa dari luar**  递送服务 / 外头打包 | Foods and Drinks:  Makanan dan minuman | Description  Ceritakan | Qty: *Kuantiti* | How many adults  Berapa orang dewasa  成年人人数 | |
|  |  | Hawkers, stalls, street food (1) *Penjaja, gerai, makanan tepi jalan*  小贩，街边摊 | Individual dishes *Barangan makanan individu*  个人餐点 | | | How many children:  *Berapa kanak-kanak*  小孩人数 | |
| 2. Outside  *Di luar*  *在外头*  *Coding the source of food among the 6 propositions below | 1. In the office *Di dalam pejabat*   办公地点 | Convenience store, supermarket, pasar mini (2) *Kedai serbaneka, pasar raya, pasar mini*  便利店，超级市场，迷你市场 |  |  |  | Guest (s) ethnicity:  *Etnik tetamu*  客人的种族 | |
|  |  | Fast – food (3)  *Makanan segera*  快餐店 |  |  |  |  |  |
|  |  |  |  |  |  | Activities during food intakes  *Aktiviti-aktiviti semasa pengambilan makanan*  用餐时的活动 | 1. Working  *Berkerja*  *工作* |
|  | 1. In a restaurant  *Di restoran*   餐馆 | Food court (4)  *Food court*  美食广场 |  |  |  |  |  |
|  |  |  |  |  |  |  | 2. Watching TV  *Menonton tv*  *看电视* |
|  |  | Mamak, nasi kandar (5) *Mamak, nasi kandar*  麻麻档，偏袒饭 | Shared food dishes *Makanan dikongsi*  共享的餐点 | | |  |  |
|  | 1. On the go *Dalam kesuntukan masa*   路途中 | Restaurant (6) *Restoran*  餐馆 |  |  |  |  | 3. On a sreen (tablet, phone, computer)  *Pada computer*  *紧盯荧幕（电脑，平板电脑，手机）* |
|  |  |  |  |  |  |  |  |
|  |  | Other place (7):  Tempat lain – lain其它地方 |  |  |  |  | 4. Meal time  *Waktu makan*  *用餐时间* |
|  | | |  |  |  |  | 5. Other distraction:  *Ganguan lain*  其它 ：  __________________ |
|  |  |  |  |  |  |  |  |

| 2 Your second food or drink intake yesterday / *Pengambilan makanan atau minuman kedua anda semalam* / 您昨天的第二份食物或饮料 | | | | | | | |
| --- | --- | --- | --- | --- | --- | --- | --- |
| Where did you have your second food or drink intake yesterday? *Dimanakah anda mengambil makanan kedua atau minuman pertama semalam?*  你昨天在哪里吃了二份食物或饮料？ | | Where did the food come from?  *Disediakan oleh siapa?*  是谁准备的食物？ | What is the name of this intake?  *Nama* Pengambilan makanan  您如何定义这一餐呢 | | | Could you describe the social context on this intake?  *Bolehkah anda menerangkan situasi anda semasa pengambilan ini?*  请说说您用餐时的情况 | |
| 1. At home  *Di Rumah*  *在家里* | 1. Your place *Tempat anda*   我家 | 1. Cooked by you  *Dimasak oleh saya*  我自己准备的 | At what time? *Pukul berapa?* 在什么时候 | | | 1. Alone  *Sendiri*  自己 | |
|  |  |  | What was the duration of this intake?  Berapa panjang masanya?  您用了多少时间 | | |  |  |
|  | 1. Friend’s place or relative’s house *Tempat Kawan atau saudaramara*   朋友或亲戚家 | 2. Cooked by friend / family  *Dimasak oleh kawan / keluarga*  朋友或家人准备的 | Could you describe the content(s) of your meal, food or drink intake?  *Bolehkah anda menerangkan kandungan makanan, atau minuman anda?*  请告诉我用餐的内容? | | | 2. With someone *  *Dengan seseorang **  与人共餐 | |
|  |  | 3. Delivery & brought from outside*  *Penghantaran & Bungkus/ Dibawa dari luar**  递送服务 / 外头打包 | Foods and Drinks:  Makanan dan minuman | Description  Ceritakan | Qty: *Kuantiti* | How many adults  Berapa orang dewasa  成年人人数 | |
|  |  | Hawkers, stalls, street food (1) *Penjaja, gerai, makanan tepi jalan*  小贩，街边摊 | Individual dishes *Barangan makanan individu*  个人餐点 | | | How many children:  *Berapa kanak-kanak*  小孩人数 | |
| 2. Outside  *Di luar*  *在外头*  *Coding the source of food among the 6 propositions below | 1. In the office *Di dalam pejabat*   办公地点 | Convenience store, supermarket, pasar mini (2) *Kedai serbaneka, pasar raya, pasar mini*  便利店，超级市场，迷你市场 |  |  |  | Guest (s) ethnicity:  *Etnik tetamu*  客人的种族 | |
|  |  | Fast – food (3)  *Makanan segera*  快餐店 |  |  |  |  |  |
|  |  |  |  |  |  | Activities during food intakes  *Aktiviti-aktiviti semasa pengambilan makanan*  用餐时的活动 | 1. Working  *Berkerja*  *工作* |
|  | 1. In a restaurant  *Di restoran*   餐馆 | Food court (4)  *Food court*  美食广场 |  |  |  |  |  |
|  |  |  |  |  |  |  | 2. Watching TV  *Menonton tv*  *看电视* |
|  |  | Mamak, nasi kandar (5) *Mamak, nasi kandar*  麻麻档，偏袒饭 | Shared food dishes *Makanan dikongsi*  共享的餐点 | | |  |  |
|  | 1. On the go *Dalam kesuntukan masa*   路途中 | Restaurant (6) *Restoran*  餐馆 |  |  |  |  | 3. On a sreen (tablet, phone, computer)  *Pada computer*  *紧盯荧幕（电脑，平板电脑，手机）* |
|  |  |  |  |  |  |  |  |
|  |  | Other place (7):  Tempat lain – lain其它地方 |  |  |  |  | 4. Meal time  *Waktu makan*  *用餐时间* |
|  | | |  |  |  |  | 5. Other distraction:  *Ganguan lain*  其它 ：  __________________ |
|  |  |  |  |  |  |  |  |

| 3 Your third food or drink intake yesterday / *Pengambilan makanan atau minumanketigaa anda semalam* / 您昨天的第三份食物或饮料 | | | | | | | |
| --- | --- | --- | --- | --- | --- | --- | --- |
| Where did you have your third food or drink intake yesterday? *Dimanakah anda mengambil makanan ketiga atau minuman pertama semalam?*  你昨天在哪里吃了第三份食物或饮料？ | | Where did the food come from?  *Disediakan oleh siapa?*  是谁准备的食物？ | What is the name of this intake?  *Nama* Pengambilan makanan  您如何定义这一餐呢 | | | Could you describe the social context on this intake?  *Bolehkah anda menerangkan situasi anda semasa pengambilan ini?*  请说说您用餐时的情况 | |
| 1. At home  *Di Rumah*  *在家里* | 1. Your place *Tempat anda*   我家 | 1. Cooked by you  *Dimasak oleh saya*  我自己准备的 | At what time? *Pukul berapa?* 在什么时候 | | | 1. Alone  *Sendiri*  自己 | |
|  |  |  | What was the duration of this intake?  Berapa panjang masanya?  您用了多少时间 | | |  |  |
|  | 1. Friend’s place or relative’s house *Tempat Kawan atau saudaramara*   朋友或亲戚家 | 2. Cooked by friend / family  *Dimasak oleh kawan / keluarga*  朋友或家人准备的 | Could you describe the content(s) of your meal, food or drink intake?  *Bolehkah anda menerangkan kandungan makanan, atau minuman anda?*  请告诉我用餐的内容? | | | 2. With someone *  *Dengan seseorang **  与人共餐 | |
|  |  | 3. Delivery & brought from outside*  *Penghantaran & Bungkus/ Dibawa dari luar**  递送服务 / 外头打包 | Foods and Drinks:  Makanan dan minuman | Description  Ceritakan | Qty: *Kuantiti* | How many adults  Berapa orang dewasa  成年人人数 | |
|  |  | Hawkers, stalls, street food (1) *Penjaja, gerai, makanan tepi jalan*  小贩，街边摊 | Individual dishes *Barangan makanan individu*  个人餐点 | | | How many children:  *Berapa kanak-kanak*  小孩人数 | |
| 2. Outside  *Di luar*  *在外头*  *Coding the source of food among the 6 propositions below | 1. In the office *Di dalam pejabat*   办公地点 | Convenience store, supermarket, pasar mini (2) *Kedai serbaneka, pasar raya, pasar mini*  便利店，超级市场，迷你市场 |  |  |  | Guest (s) ethnicity:  *Etnik tetamu*  客人的种族 | |
|  |  | Fast – food (3)  *Makanan segera*  快餐店 |  |  |  |  |  |
|  |  |  |  |  |  | Activities during food intakes  *Aktiviti-aktiviti semasa pengambilan makanan*  用餐时的活动 | 1. Working  *Berkerja*  *工作* |
|  | 1. In a restaurant  *Di restoran*   餐馆 | Food court (4)  *Food court*  美食广场 |  |  |  |  |  |
|  |  |  |  |  |  |  | 2. Watching TV  *Menonton tv*  *看电视* |
|  |  | Mamak, nasi kandar (5) *Mamak, nasi kandar*  麻麻档，偏袒饭 | Shared food dishes *Makanan dikongsi*  共享的餐点 | | |  |  |
|  | 1. On the go *Dalam kesuntukan masa*   路途中 | Restaurant (6) *Restoran*  餐馆 |  |  |  |  | 3. On a sreen (tablet, phone, computer)  *Pada computer*  *紧盯荧幕（电脑，平板电脑，手机）* |
|  |  |  |  |  |  |  |  |
|  |  | Other place (7):  Tempat lain – lain其它地方 |  |  |  |  | 4. Meal time  *Waktu makan*  *用餐时间* |
|  | | |  |  |  |  | 5. Other distraction:  *Ganguan lain*  其它 ：  __________________ |
|  |  |  |  |  |  |  |  |

| 4 Your fourth food or drink intake yesterday / *Pengambilan makanan atau minuman keempat anda semalam* / 您昨天的第四份食物或饮料 | | | | | | | |
| --- | --- | --- | --- | --- | --- | --- | --- |
| Where did you have your fourth food or drink intake yesterday? *Dimanakah anda mengambil makanan keempat atau minuman pertama semalam?*  你昨天在哪里吃了第四份食物或饮料？ | | Where did the food come from?  *Disediakan oleh siapa?*  是谁准备的食物？ | What is the name of this intake?  *Nama* Pengambilan makanan  您如何定义这一餐呢 | | | Could you describe the social context on this intake?  *Bolehkah anda menerangkan situasi anda semasa pengambilan ini?*  请说说您用餐时的情况 | |
| 1. At home  *Di Rumah*  *在家里* | 1. Your place *Tempat anda*   我家 | 1. Cooked by you  *Dimasak oleh saya*  我自己准备的 | At what time? *Pukul berapa?* 在什么时候 | | | 1. Alone  *Sendiri*  自己 | |
|  |  |  | What was the duration of this intake?  Berapa panjang masanya?  您用了多少时间 | | |  |  |
|  | 1. Friend’s place or relative’s house *Tempat Kawan atau saudaramara*   朋友或亲戚家 | 2. Cooked by friend / family  *Dimasak oleh kawan / keluarga*  朋友或家人准备的 | Could you describe the content(s) of your meal, food or drink intake?  *Bolehkah anda menerangkan kandungan makanan, atau minuman anda?*  请告诉我用餐的内容? | | | 2. With someone *  *Dengan seseorang **  与人共餐 | |
|  |  | 3. Delivery & brought from outside*  *Penghantaran & Bungkus/ Dibawa dari luar**  递送服务 / 外头打包 | Foods and Drinks:  Makanan dan minuman | Description  Ceritakan | Qty: *Kuantiti* | How many adults  Berapa orang dewasa  成年人人数 | |
|  |  | Hawkers, stalls, street food (1) *Penjaja, gerai, makanan tepi jalan*  小贩，街边摊 | Individual dishes *Barangan makanan individu*  个人餐点 | | | How many children:  *Berapa kanak-kanak*  小孩人数 | |
| 2. Outside  *Di luar*  *在外头*  *Coding the source of food among the 6 propositions below | 1. In the office *Di dalam pejabat*   办公地点 | Convenience store, supermarket, pasar mini (2) *Kedai serbaneka, pasar raya, pasar mini*  便利店，超级市场，迷你市场 |  |  |  | Guest (s) ethnicity:  *Etnik tetamu*  客人的种族 | |
|  |  | Fast – food (3)  *Makanan segera*  快餐店 |  |  |  |  |  |
|  |  |  |  |  |  | Activities during food intakes  *Aktiviti-aktiviti semasa pengambilan makanan*  用餐时的活动 | 1. Working  *Berkerja*  *工作* |
|  | 1. In a restaurant  *Di restoran*   餐馆 | Food court (4)  *Food court*  美食广场 |  |  |  |  |  |
|  |  |  |  |  |  |  | 2. Watching TV  *Menonton tv*  *看电视* |
|  |  | Mamak, nasi kandar (5) *Mamak, nasi kandar*  麻麻档，偏袒饭 | Shared food dishes *Makanan dikongsi*  共享的餐点 | | |  |  |
|  | 1. On the go *Dalam kesuntukan masa*   路途中 | Restaurant (6) *Restoran*  餐馆 |  |  |  |  | 3. On a sreen (tablet, phone, computer)  *Pada computer*  *紧盯荧幕（电脑，平板电脑，手机）* |
|  |  |  |  |  |  |  |  |
|  |  | Other place (7):  Tempat lain – lain其它地方 |  |  |  |  | 4. Meal time  *Waktu makan*  *用餐时间* |
|  | | |  |  |  |  | 5. Other distraction:  *Ganguan lain*  其它 ：  __________________ |
|  |  |  |  |  |  |  |  |

| 5 Your fifth food or drink intake yesterday / *Pengambilan makanan atau minuman kelima anda semalam* / 您昨天的第五份食物或饮料 | | | | | | | |
| --- | --- | --- | --- | --- | --- | --- | --- |
| Where did you have your fifth food or drink intake yesterday? *Dimanakah anda mengambil makanan kelima atau minuman pertama semalam?*  你昨天在哪里吃了第五份食物或饮料？ | | Where did the food come from?  *Disediakan oleh siapa?*  是谁准备的食物？ | What is the name of this intake?  *Nama* Pengambilan makanan  您如何定义这一餐呢 | | | Could you describe the social context on this intake?  *Bolehkah anda menerangkan situasi anda semasa pengambilan ini?*  请说说您用餐时的情况 | |
| 1. At home  *Di Rumah*  *在家里* | 1. Your place *Tempat anda*   我家 | 1. Cooked by you  *Dimasak oleh saya*  我自己准备的 | At what time? *Pukul berapa?* 在什么时候 | | | 1. Alone  *Sendiri*  自己 | |
|  |  |  | What was the duration of this intake?  Berapa panjang masanya?  您用了多少时间 | | |  |  |
|  | 1. Friend’s place or relative’s house *Tempat Kawan atau saudaramara*   朋友或亲戚家 | 2. Cooked by friend / family  *Dimasak oleh kawan / keluarga*  朋友或家人准备的 | Could you describe the content(s) of your meal, food or drink intake?  *Bolehkah anda menerangkan kandungan makanan, atau minuman anda?*  请告诉我用餐的内容? | | | 2. With someone *  *Dengan seseorang **  与人共餐 | |
|  |  | 3. Delivery & brought from outside*  *Penghantaran & Bungkus/ Dibawa dari luar**  递送服务 / 外头打包 | Foods and Drinks:  Makanan dan minuman | Description  Ceritakan | Qty: *Kuantiti* | How many adults  Berapa orang dewasa  成年人人数 | |
|  |  | Hawkers, stalls, street food (1) *Penjaja, gerai, makanan tepi jalan*  小贩，街边摊 | Individual dishes *Barangan makanan individu*  个人餐点 | | | How many children:  *Berapa kanak-kanak*  小孩人数 | |
| 2. Outside  *Di luar*  *在外头*  *Coding the source of food among the 6 propositions below | 1. In the office *Di dalam pejabat*   办公地点 | Convenience store, supermarket, pasar mini (2) *Kedai serbaneka, pasar raya, pasar mini*  便利店，超级市场，迷你市场 |  |  |  | Guest (s) ethnicity:  *Etnik tetamu*  客人的种族 | |
|  |  | Fast – food (3)  *Makanan segera*  快餐店 |  |  |  |  |  |
|  |  |  |  |  |  | Activities during food intakes  *Aktiviti-aktiviti semasa pengambilan makanan*  用餐时的活动 | 1. Working  *Berkerja*  *工作* |
|  | 1. In a restaurant  *Di restoran*   餐馆 | Food court (4)  *Food court*  美食广场 |  |  |  |  |  |
|  |  |  |  |  |  |  | 2. Watching TV  *Menonton tv*  *看电视* |
|  |  | Mamak, nasi kandar (5) *Mamak, nasi kandar*  麻麻档，偏袒饭 | Shared food dishes *Makanan dikongsi*  共享的餐点 | | |  |  |
|  | 1. On the go *Dalam kesuntukan masa*   路途中 | Restaurant (6) *Restoran*  餐馆 |  |  |  |  | 3. On a sreen (tablet, phone, computer)  *Pada computer*  *紧盯荧幕（电脑，平板电脑，手机）* |
|  |  |  |  |  |  |  |  |
|  |  | Other place (7):  Tempat lain – lain其它地方 |  |  |  |  | 4. Meal time  *Waktu makan*  *用餐时间* |
|  | | |  |  |  |  | 5. Other distraction:  *Ganguan lain*  其它 ：  __________________ |
|  |  |  |  |  |  |  |  |

| 6 Your sixth food or drink intake yesterday / *Pengambilan makanan atau minuman keenam anda semalam* / 您昨天的第六份食物或饮料 | | | | | | | |
| --- | --- | --- | --- | --- | --- | --- | --- |
| Where did you have your sixth food or drink intake yesterday? *Dimanakah anda mengambil makanan keenam atau minuman pertama semalam?*  你昨天在哪里吃了第六份食物或饮料？ | | Where did the food come from?  *Disediakan oleh siapa?*  是谁准备的食物？ | What is the name of this intake?  *Nama* Pengambilan makanan  您如何定义这一餐呢 | | | Could you describe the social context on this intake?  *Bolehkah anda menerangkan situasi anda semasa pengambilan ini?*  请说说您用餐时的情况 | |
| 1. At home  *Di Rumah*  *在家里* | 1. Your place *Tempat anda*   我家 | 1. Cooked by you  *Dimasak oleh saya*  我自己准备的 | At what time? *Pukul berapa?* 在什么时候  What was the duration of this intake?  Berapa panjang masanya?  您用了多少时间 | | | 1. Alone  *Sendiri*  自己 | |
|  | 1. Friend’s place or relative’s house *Tempat Kawan atau saudaramara*   朋友或亲戚家 | 2. Cooked by friend / family  *Dimasak oleh kawan / keluarga*  朋友或家人准备的 | Could you describe the content(s) of your meal, food or drink intake?  *Bolehkah anda menerangkan kandungan makanan, atau minuman anda?*  请告诉我用餐的内容? | | | 2. With someone *  *Dengan seseorang **  与人共餐 | |
|  |  | 3. Delivery & brought from outside*  *Penghantaran & Bungkus/ Dibawa dari luar**  递送服务 / 外头打包 | Foods and Drinks:  Makanan dan minuman | Description  Ceritakan | Qty: *Kuantiti* | How many adults  Berapa orang dewasa  成年人人数 | |
|  |  | Hawkers, stalls, street food (1) *Penjaja, gerai, makanan tepi jalan*  小贩，街边摊 | Individual dishes *Barangan makanan individu*  个人餐点 | | | How many children:  *Berapa kanak-kanak*  小孩人数 | |
| 2. Outside  *Di luar*  *在外头*  *Coding the source of food among the 6 propositions below | 1. In the office *Di dalam pejabat*   办公地点 | Convenience store, supermarket, pasar mini (2) *Kedai serbaneka, pasar raya, pasar mini*  便利店，超级市场，迷你市场 |  |  |  | Guest (s) ethnicity:  *Etnik tetamu*  客人的种族 | |
|  |  | Fast – food (3)  *Makanan segera*  快餐店 |  |  |  |  |  |
|  |  |  |  |  |  | Activities during food intakes  *Aktiviti-aktiviti semasa pengambilan makanan*  用餐时的活动 | 1. Working  *Berkerja*  *工作* |
|  | 1. In a restaurant  *Di restoran*   餐馆 | Food court (4)  *Food court*  美食广场 |  |  |  |  |  |
|  |  |  |  |  |  |  | 2. Watching TV  *Menonton tv*  *看电视* |
|  |  | Mamak, nasi kandar (5) *Mamak, nasi kandar*  麻麻档，偏袒饭 | Shared food dishes *Makanan dikongsi*  共享的餐点 | | |  |  |
|  | 1. On the go *Dalam kesuntukan masa*   路途中 | Restaurant (6) *Restoran*  餐馆 |  |  |  |  | 3. On a sreen (tablet, phone, computer)  *Pada computer*  *紧盯荧幕（电脑，平板电脑，手机）* |
|  |  |  |  |  |  |  |  |
|  |  | Other place (7):  Tempat lain – lain其它地方 |  |  |  |  | 4. Meal time  *Waktu makan*  *用餐时间* |
|  | | |  |  |  |  | 5. Other distraction:  *Ganguan lain*  其它 ：  __________________ |
|  |  |  |  |  |  |  |  |

| 7 Your seventh food or drink intake yesterday / *Pengambilan makanan atau minuman ketujuh anda semalam* / 您昨天的第七份食物或饮料 | | | | | | | |
| --- | --- | --- | --- | --- | --- | --- | --- |
| Where did you have your seventh food or drink intake yesterday? *Dimanakah anda mengambil makanan ketujuh atau minuman pertama semalam?*  你昨天在哪里吃了第七份食物或饮料？ | | Where did the food come from?  *Disediakan oleh siapa?*  是谁准备的食物？ | What is the name of this intake?  *Nama* Pengambilan makanan  您如何定义这一餐呢 | | | Could you describe the social context on this intake?  *Bolehkah anda menerangkan situasi anda semasa pengambilan ini?*  请说说您用餐时的情况 | |
| 1. At home  *Di Rumah*  *在家里* | 1. Your place *Tempat anda*   我家 | 1. Cooked by you  *Dimasak oleh saya*  我自己准备的 | At what time? *Pukul berapa?* 在什么时候 | | | 1. Alone  *Sendiri*  自己 | |
|  |  |  | What was the duration of this intake?  Berapa panjang masanya?  您用了多少时间 | | |  |  |
|  | 1. Friend’s place or relative’s house *Tempat Kawan atau saudaramara*   朋友或亲戚家 | 2. Cooked by friend / family  *Dimasak oleh kawan / keluarga*  朋友或家人准备的 | Could you describe the content(s) of your meal, food or drink intake?  *Bolehkah anda menerangkan kandungan makanan, atau minuman anda?*  请告诉我用餐的内容? | | | 2. With someone *  *Dengan seseorang **  与人共餐 | |
|  |  | 3. Delivery & brought from outside*  *Penghantaran & Bungkus/ Dibawa dari luar**  递送服务 / 外头打包 | Foods and Drinks:  Makanan dan minuman | Description  Ceritakan | Qty: *Kuantiti* | How many adults  Berapa orang dewasa  成年人人数 | |
|  |  | Hawkers, stalls, street food (1) *Penjaja, gerai, makanan tepi jalan*  小贩，街边摊 | Individual dishes *Barangan makanan individu*  个人餐点 | | | How many children:  *Berapa kanak-kanak*  小孩人数 | |
| 2. Outside  *Di luar*  *在外头*  *Coding the source of food among the 6 propositions below | 1. In the office *Di dalam pejabat*   办公地点 | Convenience store, supermarket, pasar mini (2) *Kedai serbaneka, pasar raya, pasar mini*  便利店，超级市场，迷你市场 |  |  |  | Guest (s) ethnicity:  *Etnik tetamu*  客人的种族 | |
|  |  | Fast – food (3)  *Makanan segera*  快餐店 |  |  |  |  |  |
|  |  |  |  |  |  | Activities during food intakes  *Aktiviti-aktiviti semasa pengambilan makanan*  用餐时的活动 | 1. Working  *Berkerja*  *工作* |
|  | 1. In a restaurant  *Di restoran*   餐馆 | Food court (4)  *Food court*  美食广场 |  |  |  |  |  |
|  |  |  |  |  |  |  | 2. Watching TV  *Menonton tv*  *看电视* |
|  |  | Mamak, nasi kandar (5) *Mamak, nasi kandar*  麻麻档，偏袒饭 | Shared food dishes *Makanan dikongsi*  共享的餐点 | | |  |  |
|  | 1. On the go *Dalam kesuntukan masa*   路途中 | Restaurant (6) *Restoran*  餐馆 |  |  |  |  | 3. On a sreen (tablet, phone, computer)  *Pada computer*  *紧盯荧幕（电脑，平板电脑，手机）* |
|  |  |  |  |  |  |  |  |
|  |  | Other place (7):  Tempat lain – lain其它地方 |  |  |  |  | 4. Meal time  *Waktu makan*  *用餐时间* |
|  | | |  |  |  |  | 5. Other distraction:  *Ganguan lain*  其它 ：  __________________ |
|  |  |  |  |  |  |  |  |

| 8 Your eighth food or drink intake yesterday / *Pengambilan makanan atau minuman kelapan anda semalam* / 您昨天的第八份食物或饮料 | | | | | | | |
| --- | --- | --- | --- | --- | --- | --- | --- |
| Where did you have your eighth food or drink intake yesterday? *Dimanakah anda mengambil makanan kelapan atau minuman pertama semalam?*  你昨天在哪里吃了第八份食物或饮料？ | | Where did the food come from?  *Disediakan oleh siapa?*  是谁准备的食物？ | What is the name of this intake?  *Nama* Pengambilan makanan  您如何定义这一餐呢 | | | Could you describe the social context on this intake?  *Bolehkah anda menerangkan situasi anda semasa pengambilan ini?*  请说说您用餐时的情况 | |
| 1. At home  *Di Rumah*  *在家里* | 1. Your place *Tempat anda*   我家 | 1. Cooked by you  *Dimasak oleh saya*  我自己准备的 | At what time? *Pukul berapa?* 在什么时候 | | | 1. Alone  *Sendiri*  自己 | |
|  |  |  | What was the duration of this intake?  Berapa panjang masanya?  您用了多少时间 | | |  |  |
|  | 1. Friend’s place or relative’s house *Tempat Kawan atau saudaramara*   朋友或亲戚家 | 2. Cooked by friend / family  *Dimasak oleh kawan / keluarga*  朋友或家人准备的 | Could you describe the content(s) of your meal, food or drink intake?  *Bolehkah anda menerangkan kandungan makanan, atau minuman anda?*  请告诉我用餐的内容? | | | 2. With someone *  *Dengan seseorang **  与人共餐 | |
|  |  | 3. Delivery & brought from outside*  *Penghantaran & Bungkus/ Dibawa dari luar**  递送服务 / 外头打包 | Foods and Drinks:  Makanan dan minuman | Description  Ceritakan | Qty: *Kuantiti* | How many adults  Berapa orang dewasa  成年人人数 | |
|  |  | Hawkers, stalls, street food (1) *Penjaja, gerai, makanan tepi jalan*  小贩，街边摊 | Individual dishes *Barangan makanan individu*  个人餐点 | | | How many children:  *Berapa kanak-kanak*  小孩人数 | |
| 2. Outside  *Di luar*  *在外头*  *Coding the source of food among the 6 propositions below | 1. In the office *Di dalam pejabat*   办公地点 | Convenience store, supermarket, pasar mini (2) *Kedai serbaneka, pasar raya, pasar mini*  便利店，超级市场，迷你市场 |  |  |  | Guest (s) ethnicity:  *Etnik tetamu*  客人的种族 | |
|  |  | Fast – food (3)  *Makanan segera*  快餐店 |  |  |  |  |  |
|  |  |  |  |  |  | Activities during food intakes  *Aktiviti-aktiviti semasa pengambilan makanan*  用餐时的活动 | 1. Working  *Berkerja*  *工作* |
|  | 1. In a restaurant  *Di restoran*   餐馆 | Food court (4)  *Food court*  美食广场 |  |  |  |  |  |
|  |  |  |  |  |  |  | 2. Watching TV  *Menonton tv*  *看电视* |
|  |  | Mamak, nasi kandar (5) *Mamak, nasi kandar*  麻麻档，偏袒饭 | Shared food dishes *Makanan dikongsi*  共享的餐点 | | |  |  |
|  | 1. On the go *Dalam kesuntukan masa*   路途中 | Restaurant (6) *Restoran*  餐馆 |  |  |  |  | 3. On a sreen (tablet, phone, computer)  *Pada computer*  *紧盯荧幕（电脑，平板电脑，手机）* |
|  |  |  |  |  |  |  |  |
|  |  | Other place (7):  Tempat lain – lain其它地方 |  |  |  |  | 4. Meal time  *Waktu makan*  *用餐时间* |
|  | | |  |  |  |  | 5. Other distraction:  *Ganguan lain*  其它 ：  __________________ |
|  |  |  |  |  |  |  |  |

| 9 Your ninth food or drink intake yesterday / *Pengambilan makanan atau minuman kesembilan anda semalam* / 您昨天的第九份食物或饮料 | | | | | | | |
| --- | --- | --- | --- | --- | --- | --- | --- |
| Where did you have your ninth food or drink intake yesterday? *Dimanakah anda mengambil makanan kesembilan atau minuman pertama semalam?*  你昨天在哪里吃了第九份食物或饮料？ | | Where did the food come from?  *Disediakan oleh siapa?*  是谁准备的食物？ | What is the name of this intake?  *Nama* Pengambilan makanan  您如何定义这一餐呢 | | | Could you describe the social context on this intake?  *Bolehkah anda menerangkan situasi anda semasa pengambilan ini?*  请说说您用餐时的情况 | |
| 1. At home  *Di Rumah*  *在家里* | 1. Your place *Tempat anda*   我家 | 1. Cooked by you  *Dimasak oleh saya*  我自己准备的 | At what time? *Pukul berapa?* 在什么时候 | | | 1. Alone  *Sendiri*  自己 | |
|  |  |  | What was the duration of this intake?  Berapa panjang masanya?  您用了多少时间 | | |  |  |
|  | 1. Friend’s place or relative’s house *Tempat Kawan atau saudaramara*   朋友或亲戚家 | 2. Cooked by friend / family  *Dimasak oleh kawan / keluarga*  朋友或家人准备的 | Could you describe the content(s) of your meal, food or drink intake?  *Bolehkah anda menerangkan kandungan makanan, atau minuman anda?*  请告诉我用餐的内容? | | | 2. With someone *  *Dengan seseorang **  与人共餐 | |
|  |  | 3. Delivery & brought from outside*  *Penghantaran & Bungkus/ Dibawa dari luar**  递送服务 / 外头打包 | Foods and Drinks:  Makanan dan minuman | Description  Ceritakan | Qty: *Kuantiti* | How many adults  Berapa orang dewasa  成年人人数 | |
|  |  | Hawkers, stalls, street food (1) *Penjaja, gerai, makanan tepi jalan*  小贩，街边摊 | Individual dishes *Barangan makanan individu*  个人餐点 | | | How many children:  *Berapa kanak-kanak*  小孩人数 | |
| 2. Outside  *Di luar*  *在外头*  *Coding the source of food among the 6 propositions below | 1. In the office *Di dalam pejabat*   办公地点 | Convenience store, supermarket, pasar mini (2) *Kedai serbaneka, pasar raya, pasar mini*  便利店，超级市场，迷你市场 |  |  |  | Guest (s) ethnicity:  *Etnik tetamu*  客人的种族 | |
|  |  | Fast – food (3)  *Makanan segera*  快餐店 |  |  |  |  |  |
|  |  |  |  |  |  | Activities during food intakes  *Aktiviti-aktiviti semasa pengambilan makanan*  用餐时的活动 | 1. Working  *Berkerja*  *工作* |
|  | 1. In a restaurant  *Di restoran*   餐馆 | Food court (4)  *Food court*  美食广场 |  |  |  |  |  |
|  |  |  |  |  |  |  | 2. Watching TV  *Menonton tv*  *看电视* |
|  |  | Mamak, nasi kandar (5) *Mamak, nasi kandar*  麻麻档，偏袒饭 | Shared food dishes *Makanan dikongsi*  共享的餐点 | | |  |  |
|  | 1. On the go *Dalam kesuntukan masa*   路途中 | Restaurant (6) *Restoran*  餐馆 |  |  |  |  | 3. On a sreen (tablet, phone, computer)  *Pada computer*  *紧盯荧幕（电脑，平板电脑，手机）* |
|  |  |  |  |  |  |  |  |
|  |  | Other place (7):  Tempat lain – lain其它地方 |  |  |  |  | 4. Meal time  *Waktu makan*  *用餐时间* |
|  | | |  |  |  |  | 5. Other distraction:  *Ganguan lain*  其它 ：  __________________ |
|  |  |  |  |  |  |  |  |

| 10 Your tenth food or drink inatake yesterday / *Pengambilan makanan atau minuman kesepuluh anda semalam* / 您昨天的第十份食物或饮料 | | | | | | | |
| --- | --- | --- | --- | --- | --- | --- | --- |
| Where did you have your tenth food or drink intake yesterday? *Dimanakah anda mengambil makanan kesepuluha atau minuman pertama semalam?*  你昨天在哪里吃了第十份食物或饮料？ | | Where did the food come from?  *Disediakan oleh siapa?*  是谁准备的食物？ | What is the name of this intake?  *Nama* Pengambilan makanan  您如何定义这一餐呢 | | | Could you describe the social context on this intake?  *Bolehkah anda menerangkan situasi anda semasa pengambilan ini?*  请说说您用餐时的情况 | |
| 1. At home  *Di Rumah*  *在家里* | 1. Your place *Tempat anda*   我家 | 1. Cooked by you  *Dimasak oleh saya*  我自己准备的 | At what time? *Pukul berapa?* 在什么时候 | | | 1. Alone  *Sendiri*  自己 | |
|  |  |  | What was the duration of this intake?  Berapa panjang masanya?  您用了多少时间 | | |  |  |
|  | 1. Friend’s place or relative’s house *Tempat Kawan atau saudaramara*   朋友或亲戚家 | 2. Cooked by friend / family  *Dimasak oleh kawan / keluarga*  朋友或家人准备的 | Could you describe the content(s) of your meal, food or drink intake?  *Bolehkah anda menerangkan kandungan makanan, atau minuman anda?*  请告诉我用餐的内容? | | | 2. With someone *  *Dengan seseorang **  与人共餐 | |
|  |  | 3. Delivery & brought from outside*  *Penghantaran & Bungkus/ Dibawa dari luar**  递送服务 / 外头打包 | Foods and Drinks:  Makanan dan minuman | Description  Ceritakan | Qty: *Kuantiti* | How many adults  Berapa orang dewasa  成年人人数 | |
|  |  | Hawkers, stalls, street food (1) *Penjaja, gerai, makanan tepi jalan*  小贩，街边摊 | Individual dishes *Barangan makanan individu*  个人餐点 | | | How many children:  *Berapa kanak-kanak*  小孩人数 | |
| 2. Outside  *Di luar*  *在外头*  *Coding the source of food among the 6 propositions below | 1. In the office *Di dalam pejabat*   办公地点 | Convenience store, supermarket, pasar mini (2) *Kedai serbaneka, pasar raya, pasar mini*  便利店，超级市场，迷你市场 |  |  |  | Guest (s) ethnicity:  *Etnik tetamu*  客人的种族 | |
|  |  | Fast – food (3)  *Makanan segera*  快餐店 |  |  |  |  |  |
|  |  |  |  |  |  | Activities during food intakes  *Aktiviti-aktiviti semasa pengambilan makanan*  用餐时的活动 | 1. Working  *Berkerja*  *工作* |
|  | 1. In a restaurant  *Di restoran*   餐馆 | Food court (4)  *Food court*  美食广场 |  |  |  |  |  |
|  |  |  |  |  |  |  | 2. Watching TV  *Menonton tv*  *看电视* |
|  |  | Mamak, nasi kandar (5) *Mamak, nasi kandar*  麻麻档，偏袒饭 | Shared food dishes *Makanan dikongsi*  共享的餐点 | | |  |  |
|  | 1. On the go *Dalam kesuntukan masa*   路途中 | Restaurant (6) *Restoran*  餐馆 |  |  |  |  | 3. On a sreen (tablet, phone, computer)  *Pada computer*  *紧盯荧幕（电脑，平板电脑，手机）* |
|  |  |  |  |  |  |  |  |
|  |  | Other place (7):  Tempat lain – lain其它地方 |  |  |  |  | 4. Meal time  *Waktu makan*  *用餐时间* |
|  | | |  |  |  |  | 5. Other distraction:  *Ganguan lain*  其它 ：  __________________ |
|  |  |  |  |  |  |  |  |

| Q11 | *Type of food*  食物类型  Jenis Makanan | *How frequent each food was taken (Fill in one of the columns only)*  您通常多久吃这类食物 (只填写其中一格)  Berapa kali kekerapan pengambilan dalam (Isikan dalam salah satu kolum sahaja) | | | |
| --- | --- | --- | --- | --- | --- |
| no |  | a.  *Daily*  *每一天*  Sehari | b.  *Weekly*  每周  Seminggu | c.  *Monthly*  *每月*  Sebulan | d.  Seldom  几乎没有 Jarang |
| *Cereals and cereals product* / 谷类 / Bijirin dan hasil bijirin | | | | | |
| 1 | *White rice*  *米饭*  Nasi putih |  |  |  |  |
| 2 | *Flavoured rice （Fried rice, Nasi Beriyina etc）*  有味道的米饭 (炒饭，印度香米饭等等)  Nasi berperisa (Nasi briyani, nasi goreng dsb.) |  |  |  |  |
| 3 | *Noodles*  黄面条  Mee kuning/ Mee siput/ Mee segera/ |  |  |  |  |
| 4 | Mihun / Kueh teow/ Laksa/ Laksam/ *Loh shi fun*  *米粉* /*果条*  / *拉沙*  / *老鼠粉* |  |  |  |  |
| 5 | Sagu/ ambuyat/ linut  *Sago/ambuyat/linut*  *西米* |  |  |  |  |
| 6 | *Bread (white, wholemeal, buns, etc.)*  *面包 ( 白，全麦，包子等 )*  *Roti (putih, berserat, bun)* |  |  |  |  |
| 7 | Roti canai (termasuk roti telur, roti sardin, roti bawang, roti pisang, murtabak)  印度煎饼 （包括鸡蛋煎饼，萨丁煎饼，大葱煎饼，香蕉煎饼，馅料煎饼） |  |  |  |  |
| 8 | *Corn*  玉蜀黍  Jagung |  |  |  |  |

| no | *Type of food*  食物类型  Jenis Makanan | *How frequent each food was taken (Fill in one of the columns only)*  您通常多久吃这类食物 (只填写其中一格)  Berapa kali kekerapan pengambilan dalam (Isikan dalam salah satu kolum sahaja) | | | |
| --- | --- | --- | --- | --- | --- |
|  |  | a.  *Daily*  *每一天*  Sehari | b.  *Weekly*  每周  Seminggu | c.  *Monthly*  *每月*  Sebulan | d.  Seldom  几乎没有 Jarang |
| *Meat and meat product*肉类Daging dan hasil daging | | | | | |
| 9 | *Chicken & Duck*  家禽类  Ayam dan Itik |  |  |  |  |
| 10 | *Meat* (beef/buffalo)  红肉(牛肉 / 羊肉)  Daging (Lembu/ Kerbau) |  |  |  |  |
| 11 | *Pork*  猪肉  Babi |  |  |  |  |
| 12 | *Other types of meat*  *其它*  Jenis Daging yang lain  ___________________ |  |  |  |  |
| *Fish ans seafoods*鱼与海鲜Ikan dan makanan laut | | | | | |
| 13 | *Salted & Fresh Water fish*  海鱼与河鱼  Ikan laut & ikan air tawar |  |  |  |  |
| 14 | *Fish crackers*  炸鱼饼  Keropok kan |  |  |  |  |
| *Eggs*蛋类Telur | | | | | |
| 15 | Eggs (*Hen eggs Sunny, omelet, boiled, with chilies or herbs)*  蛋类 ( 各式各类的煮蛋)  Telur  (mata kerbau, telur dadar, telur rebus, telur masak sambal, telur pindang) |  |  |  |  |
| *Fast Food* 快餐 Makanan segera | | | | | |
| 16 | Burger  (except egg & veggie burger)  汉堡 ( 除了素汉堡与蛋汉堡)  Burger (Kecuali Benjol atau burger Sayuran) |  |  |  |  |
| 17 | *Fried chicken*  炸鸡  Ayam goreng |  |  |  |  |

| no | *Type of food*  食物类型  Jenis Makanan | *How frequent each food was taken (Fill in one of the columns only)*  您通常多久吃这类食物 (只填写其中一格)  Berapa kali kekerapan pengambilan dalam (Isikan dalam salah satu kolum sahaja) | | | |
| --- | --- | --- | --- | --- | --- |
|  |  | a.  *Daily*  *每一天*  Sehari | b.  *Weekly*  每周  Seminggu | c.  *Monthly*  *每月*  Sebulan | d.  Seldom  几乎没有 Jarang |
| *Legumes and legumes 豆类与其完成品 products* Kekacang dan hasilnya | | | | | |
| 18 | *Legumes*  豆类 (绿豆，蝉豆，鹰嘴豆，红豆)  Kekacang  (kacang hijau, kacang parang, kacang kuda, kacang merah dsb.) |  |  |  |  |
| 19 | *Groundnuts*  花生  Kacang tanah |  |  |  |  |
| 20 | *Soya Bean Curd*  豆腐花 / 豆腐  Taufufa & Tauf*u* |  |  |  |  |
| 21 | *Fermented soy beans*  天贝  Tempe |  |  |  |  |
| *Milk and milk products* 牛奶与奶制品Susu dan hasil tenusu | | | | | |
| 22 | *Milk (including liquid/powder; low/full fat; cow & goat)*  牛奶 ( 包括液状，粉转，低脂肪 / 全脂 ； 牛奶，羊奶)  Susu(termasukcecair/tepung, rendah/penuh lemak, susu lembu/susu kambing |  |  |  |  |
| 23 | Milk shake, smoothies, ice cream, yogurt  奶昔，流沙，冰淇淋，优格  Susu shake,smoothies, Ais krim, yogurt |  |  |  |  |

| no | *Type of food*  食物类型  Jenis Makanan | *How frequent each food was taken (Fill in one of the columns only)*  您通常多久吃这类食物 (只填写其中一格)  Berapa kali kekerapan pengambilan dalam (Isikan dalam salah satu kolum sahaja) | | | |
| --- | --- | --- | --- | --- | --- |
|  |  | a.  *Daily*  *每一天*  Sehari | b.  *Weekly*  每周  Seminggu | c.  *Monthly*  *每月*  Sebulan | d.  Seldom  几乎没有 Jarang |
| *Vegetables 蔬菜*  Sayur – Sayuran | | | | | |
| 24 | *Green leafy vegetables*  青色蔬菜  Sayuran berdaun hijau (bayam, kangkung, kailan dsb.) |  |  |  |  |
| 25 | *Ladies finger*  羊角豆  Bendi |  |  |  |  |
| 26 | *Other type of legumes*  其它蔬菜豆类  Sayur kekacang lain (kacang panjang, kacang buncis, kacang botol dsb.) |  |  |  |  |
| 27 | *Bean sprout*  豆芽  Taugeh/ |  |  |  |  |
| 28 | *Tubers (potatoes, sweet potatoes, yam)*  *薯苓类*  Sayuran berubi (kentang, keladi, keledek)/ |  |  |  |  |
| 29 | *Cabbages*  包菜  Sayuran kobis (kobis bulat/ brokoli, kobis cina, bunga kobis)/ |  |  |  |  |
| 30 | *Fruit vegetables (luffa, pumpkin, cucumber/ baby corn)*  *水果蔬菜 ( 丝瓜，南瓜，黄瓜，小玉蜀黍 )*  Sayuran berbuah lain (petola/ labu/ timun/ putik jagung)/ |  |  |  |  |

| no | *Type of food*  食物类型  Jenis Makanan | *How frequent each food was taken (Fill in one of the columns only)*  您通常多久吃这类食物 (只填写其中一格)  Berapa kali kekerapan pengambilan dalam (Isikan dalam salah satu kolum sahaja) | | | |
| --- | --- | --- | --- | --- | --- |
|  |  | a.  *Daily*  *每一天*  Sehari | b.  *Weekly*  每周  Seminggu | c.  *Monthly*  *每月*  Sebulan | d.  Seldom  几乎没有 Jarang |
| *Drinks* 饮料Minuman | | | | | |
| 31 | *Soy milk*  豆奶  Susu kacang soya |  |  |  |  |
| 32 | Chocolate Malt Drink  巧克力麦料饮品  Minuman Bijirin Chocolate |  |  |  |  |
| *Flavours配料*Perencah / Peresa | | | | | |
| 33 | *Soy sauce*  酱油  Kicap |  |  |  |  |

| **Section D: Cooking practices** |
| --- |

| Q12 | Generally, who is cooking in your household? (Choose one answer)  通常谁为您家人做饭？  *Secara umumnya, siapakah yang masak di rumah anda?* | SA | Route |
| --- | --- | --- | --- |
|  | Myself / 我自己 / *Sendiri* | 1 | Continue |
|  | My wife / 我的妻子 / *Isteri Saya* | 2 |  |
|  | My husband / 我的丈夫 / *Suami Saya* | 3 |  |
|  | My Grandparent(s) / 公公婆婆 / *Datuk nenek* | 4 |  |
|  | My Friend / 朋友 */ Kawan-kawan* | 5 |  |
|  | My mother / 我的母亲 / *Emak Saya* | 6 |  |
|  | A maid / A helper / 女佣 / *Pembantu rumah* | 7 |  |
|  | I hardly cook at home / 我很少在家里做饭 / *Saya jarang memasak di rumah* | 8 |  |
|  | Others / 其 他 / lain-lain | 9 |  |

| **Section E : Representations of food** |
| --- |

| Q13 | Which of the following statement do you feel closer to? (Choose one answer)  您认为以下那一项最符合您的想法？  *Antara penyataan berikut yang manakah anda rasakan yang sesuai dengan anda*  ***[SHOW CARD]*** | SA | Route |
| --- | --- | --- | --- |
|  | Food must be first of all a need  民以食为天  *Makanan adalah satu keperluan* | 1 | Continue |
|  | Food must be first of all shared with someone  食物是应该与他人分享的  *Makanan sepatutnya dikongsi dengan seseorang* | 2 |  |
|  | Food must be first of all a pleasure  食物是应该被享受的  *Makanan mestilah seronok* | 3 |  |
|  | Food must first of all prevent health problems  食物是应该以养生为主  *Makanan paling utamanya dapat mencegah masalah kesihatan* | 4 |  |
|  | Other description  其他 -------------------  *lain-lain* | 5 |  |

| Q14 | Could you tell me 2 dishes that best represent Malaysian food?  请您说出2样最能代表马来西亚食物的菜肴  *Boleh beritahu saya 2 hidangan yang terbaik yang mewakili makanan Malaysia?* | MA | Route |
| --- | --- | --- | --- |
|  |  | 1 | Continue |
|  |  | 2 |  |

| Q14bis | If Nasi Lemak was selected as a dish to present Malaysian Identity, would you support the idea?  如果椰浆饭被选为最具马来西亚彩色的菜肴，您会赞成吗？  Jika nasi lemak dipilih sebagai hidangan melambangkan identity Malaysia, adakah anda bersetuju? | SA | Route |
| --- | --- | --- | --- |
|  | Strongly Agree  非常赞成  Paling Bersetuju | 1 | Continue |
|  | Agree  赞成  Bersetuju | 2 |  |
|  | Disagree  不赞成  Tidak bersetuju | 3 |  |
|  | Strongly Disagree  非常不赞成  Paling Tidak Bersetuju | 4 |  |

| Q15 | What does “Eating Well” mean to you?  您认为“吃得好”是什么定义呢？  *Pada pendapat anda, apakah yang dimaksudken dengan “Makan Secara baik? (TUNJUK KAD)*  ***[SHOW CARD]*** | 1^st^  (SA) | 2^nd^  (SA) | Route |
| --- | --- | --- | --- | --- |
|  | Health  吃得健康  *Kesihatan* | 1 | 1 | Continue |
|  | Pleasure  高兴地吃  *Kesukaan* | 2 | 2 |  |
|  | Togetherness  与伙伴大吃大喝  *Makan bersama* | 3 | 3 |  |
|  | Fill the stomach  填饱肚子 *Mengenyangkan* | 4 | 4 |  |
|  | Traditions  吃得传统 *Tradisi* | 5 | 5 |  |
|  | Gives Energy  力气  Memberikan Tenaga | 6 | 6 |  |
|  | Others  其它 *Lain-lain* | 7 | 7 |  |

| **Section F: Health and risk issues** |
| --- |

| Q16 | According to you, what are the 2 essential foodstuffs (food items, meals, ingredients, drinks…) that are essentials and beneficial to the health?  在您看来，有哪2种食品 ( 食物，成分，饮料。。。)对使身体健康来说是不可缺少的  *Pada pendapat anda, apakah 2 bahan makanan yang paling bermanfaat untuk kesihatan?* | MA | Route |
| --- | --- | --- | --- |
|  |  | 1 | Continue |
|  |  | 2 |  |

| Q17 | According to you, what are the 2 essential foodstuffs to reduce to be in good health?  在您看来， 又有哪2种食品对使身体健康来说是必须要减少的  *Pada pendapat anda, apakah 2 bahan makanan yang perlu dikurangkan untuk kesihatan yang baik?* | MA | Route |
| --- | --- | --- | --- |
|  |  | 1 | Continue |
|  |  | 2 |  |

| Q18 | Which of the following risks scare you the most? Identify your top 3 risks  您最担心以下哪一些风险呢？请说出令您最提心吊胆的3项风险  *Diantara risiko berikut, yang mana paling menakutkan anda? Namakan 3 tiga risiko yang tertinggi*  ***[SHOW CARD]*** | | | |
| --- | --- | --- | --- | --- |
|  |  | 1^st^ | 2^nd^ | 3^rd^ |
| a | Pesticides on agricultural products  农作物上的农药  *Racun perosak produk pertanian* | 1 | 1 | 1 |
| b | Genetically Modify Organism (GMO)  基因改造食物  *Organisma terubah suai secara genetic* | 2 | 2 | 2 |
| c | Contamination by Pollutant  被污染物所污染  *Pencemaran oleh Pencemar* | 3 | 3 | 3 |
| d | Colouring or preservatives  色素和防腐剂  *Pewarna atau pengawet* | 4 | 4 | 4 |
| e | Germs or bacteria in Food  食物里的细菌或病菌  *Kuman atau bacteria dalam makanan* | 5 | 5 | 5 |
| f | Food epidemic (i.e. Bird flu)  通过食物传播的流行性疫病  （禽流感等等）  *Kuman atau bacteria dalam makanan ( Bird flu…)* | 6 | 6 | 6 |
| g | Unbalance diet to fat or too much sugar  不均衡的饮食脂肪或糖分过多  *Diet tidak seimbang terhadap lemak atau terlalu banyak gula* | 7 | 7 | 7 |
| h | Expired food  过期的食物  *Makanan tamat tempoh* | 8 | 8 | 8 |
| i | Food allergens (i.e. nuts….)  食物过敏（对花生敏感）  *Alahan Makanan (Kacang dan lain-lain)* | 9 | 9 | 9 |

| **Section G: Ethnicity indicators** |
| --- |

| Q19a | What is your cultural identity? (3 words)  请告诉我，您本身的文化特色。（3句）  *Sila beritahu kami bagaimana anda tentukan identiti budaya anda? (3 perkataan)* | MA | Route |
| --- | --- | --- | --- |
|  |  | 1 | Continue |
|  |  | 2 |  |
|  |  | 3 |  |

| Q19b | Please rank 3 best words from below that how you defined your identity in Malaysia?  请从以下的字句排出3最能代表您在马来西亚的身份  *Sila aturkan 3 perkataan dibawah yang paling menggambarkan identity anda di Malaysia.*  ***[SHOW CARD]*** | MA | Route |
| --- | --- | --- | --- |
|  |  | 1 | Continue |
|  |  | 2 |  |
|  |  | 3 |  |

| Q20 | Could you indicate the ethnicity / race of both your parents and grand – parents in the following chart?  您能在下面的图表中指出您的父母及祖父母的种族吗？  ***Bolehkah anda menunjukkan etnik ibu bapa anda dan datuk nenek dalam carta berikut***  *[SHOW CARD]* |
| --- | --- |

| Q21 | What languages or dialects do you speak?  您通常都用哪一种语言和方言交谈呢？  *Apakah bahasa atau dialek anda ?* | MA | Route |
| --- | --- | --- | --- |
|  | Malay / 马来语 / *Bahasa Melayu* | 1 | Continue |
|  | English / 英语 / *Bahasa Inggeris* | 2 |  |
|  | Arabic / 阿拉伯语 / *Bahasa Arab* | 3 |  |
|  | Mandarin / *华语 / Bahasa Cina* | 4 |  |
|  | Cantonese / 广东话 / *Bahasa Kantonis* | 5 |  |
|  | Hokkien / 福建话 / Bahasa Hokkien | 6 |  |
|  | Hakka / 客家话 / Bahasa Hakka | 7 |  |
|  | Tamil / 淡米尔语 / *Bahasa Tamil* | 8 |  |
|  | Hindi / 印度话 / *Bahasa Hindi* | 9 |  |
|  | Other(s): / 其他 / *Lain-lain___________________________* | 10 |  |

| Q22a | What is your religion? / 您的宗教信仰? / *Apakah agama anda?* | MA | Route |
| --- | --- | --- | --- |
|  | Muslim / 穆斯林 / *Islam* | 1 | Continue |
|  | Hindu / 兴度教 / *Hindu* | 2 |  |
|  | Buddhist / 佛教 / *Buddha* | 3 |  |
|  | Christian / 基督教 / *Kristian* | 4 |  |
|  | Taoism / 道教 / *Tao* | 5 |  |
|  | Other: / 其他 / *Lain-lain* | 6 |  |
|  | No religion / 没有信仰 / *Tiada agama* | 7 |  |
|  | Decline to answer (Do not read) / 拒绝 *Enggan menjawab* | 8 |  |

| Q22b | Are you a convert? / 您曾经转换信仰吗？/ Pernahkah anda bertukar agama? | SA | Route |
| --- | --- | --- | --- |
|  | Yes / 有 / Ya | 1 | Go to Q22c |
|  | No / 没有 / Tidak | 2 | Go to Q23 |

| Q22c | If YES, what was your previous religion and what is your religion now?  如果有的话，那您是从什么信仰转换至现在的信仰呢？  Jika ia (ya), apakah agama anda sebelum ini dan apakah agama anda sekarang? | SA |  |
| --- | --- | --- | --- |
|  |  | Before  曾经  *Sebelum* | Continue |

| Q23 | We would like to understand the influence of religion in your food habits. Would you describe yourself as  我们希望了解宗教信仰对您选择食物时的影响，您会说您是。  Kami ingin mengetahui apakah kesan agama terhadap tabiat makanan anda. *Anda adalah seorang yang:*  ***[SHOW CARD]*** | SA | Route |
| --- | --- | --- | --- |
|  | Very religious  非常虔诚  *Kuat beragama* | 1 | Continue |
|  | Moderately religious  适度的虔诚  *Sederhana beragama* | 2 |  |
|  | Lightly religious  有一点虔诚  *Tidak kuat beragama* | 3 |  |
|  | Not religious at all  完全不虔诚  *Tidak beragama* | 4 |  |
|  | Decline to answer (Do not read)  拒绝  *Enggan menjawab* | 5 |  |

| **Section H: Socio – Demographic indicators** |
| --- |

| Q24 | How tall are you without your shoes on?  您没有穿鞋的高度是？  *Apakah ketinggian anda tanpa kasut?* | SA | Route |
| --- | --- | --- | --- |
|  |  | cm | Continue |

| Q25 | How much do you weigh without your shoes on?  您没有穿鞋的体重是？  *Berapakah berat badan anda tanpa kasut?* | SA | Route |
| --- | --- | --- | --- |
|  |  | kg | Continue |

| Q26 | What is your occupation? (Do not read first, if retired, the latest job)  您的职业是？  *Apakah perkerjaan anda?* | | 1st | 2nd | Route |
| --- | --- | --- | --- | --- | --- |
|  | **1^st^ Answer:** |  |  |  | Continue |
|  | **2^nd^ Answer:** |  |  |  |  |
|  | Decline to answer拒绝*Enggan menjawab* (do not read first) | | 99 | 99 |  |

| Q27 | What is your father’s last occupation?  If a single mother raised the respondent: What is/was your mother’s occupation?  您的父亲最后是从事哪一个行业;如果您是来自单亲家庭，您母亲的职业是？)  Pekerjaan Bapa anda perkerjaan terakhir? Jika seorang ibu tunggal menaikkan responden: Apakah / pekerjaan ibu anda? | | SA | Route |
| --- | --- | --- | --- | --- |
|  | **Answer:**  If several, the one for the longest period of time)  如果有多份工作，请说出从事最久的职业  Jika beberapa, sila namakan perkejaan untuk masa yang paling lama |  | | Continue |
|  | Decline to answer拒绝*Enggan menjawab* (do not read first) | | 99 |  |

| Q28 | What is the highest level of education that you have completed?  您所完成的最高学历？  *Apakah tahap pendidikan tertinggi yang anda telah lengkapkan?* | SA | Route |
| --- | --- | --- | --- |
|  | No formal education没有受过正统的教育*Tiada pendidikan rasmi* | 1 | Continue |
|  | Primary school小学*Sekolah rendah* | 2 |  |
|  | Lower secondary school / SRP初中 / SRP  *Sekolah Menengah rendahi*  / *SRP* | 3 |  |
|  | Upper secondary school SPM / O Level高中 SPM / O Level  *Sekolah Menengah tinggi SPM* / O Level | 4 |  |
|  | Marticulation / Form 6 STPM / A Level中六 STPM / A Level  *Matrikulasi / Form 6 STPM* / *A Level* | 5 |  |
|  | College / University学院 ／ 大学*Kolej / Universiti* | 6 |  |
|  | Other : 其他*Lain-lain* | 7 |  |
|  | Decline to answer拒绝*Enggan menjawab* (do not read first) | 8 |  |

| Q29a | What is your marital status? / 您的婚姻状况 / *Apakah status perkahwinan anda?* | SA | Route |
| --- | --- | --- | --- |
|  | Single or never married / 单身或没有结婚 / *Bujang atau tidak pernah berkahwin* | 1 | Go to Q30 |
|  | Married in monogamous marriage / 一夫一妻制的婚姻 / *Berkahwin dalam perkahwinan monogami* | 2 | Continue |
|  | Married in polygamous marriage / 一夫多妻制的婚姻 / *Berkahwin dalam perkahwinan poligami* | 3 |  |
|  | Living in as married (or cohabitation) / 同居 / *Tinggal sebagai berkahwin* | 4 |  |
|  | Widowed / 鳏夫 或 寡妇 / *Janda/duda* | 5 |  |
|  | Separated or married but separated / 分居 /  *berkahwin tapi tinggal berasingan* | 6 |  |
|  | Not living with legal spouse / 结婚了但没有与合法伴侣住在一起  *Tidak tinggal dengan pasangan yang sah* | 7 |  |
|  | Divorced / 离婚 / *Bercerai* | 8 |  |
|  | Decline to answer / 拒绝 */ Enggan menjawab* | 9 | Go to Q30 |

| Q29b | Could you indicate the ethnicity / race of your spouse? (do not read first)  您可以告诉我您的配偶的种族吗？  Boleh anda nyatakan etnik/bangsa isteri anda? (jangan baca dahulu) | MA | Route |
| --- | --- | --- | --- |
|  | Non – Malay Bumiputra / 非巫族土著 / Bumiputera bukan Melayu | 1 | Continue |
|  | Malay / 巫族 / *Melayu* | 2 |  |
|  | Indian / 印族 / *India* | 3 |  |
|  | Chinese / 华族 / *Cina* | 4 |  |
|  | Others / 其他 / *Lain-lain* | 5 |  |

| Q30 | How many children do you have / 您拥有多少个小孩？ / *Berapa kanak-kanak anda mempunyai?* | SA | Route |
| --- | --- | --- | --- |
|  |  |  | Continue |

| Q31 | How many family members (including yourself live together with you under the same roof?  包括自己，您与多少个家人成员生活在一起？  *Berapa ahli keluarga (termausk diri sendiri) tinggal bersama dengan anda?* | SA | Route |
| --- | --- | --- | --- |
|  |  |  | Continue |

| Q32 | We would like to know your average monthly household income, based on your wages, salaries, pensions, dividends and other income before taxes and other education.  What is the absolute value?  我们只想知道您的家庭平均总收入大约是多少 ，请算算所有家人的工资，薪金，退休金，红利支付和其他收入。在所有的税收前的您的家庭收入是多少。  *Kami ingin mengetahui secara puratanya, upah bulanan anda dengan mengunakan skala yang berikut, mengambilkira upah, gaji, pencen, dividen dan pendapatan lain sebelum cukai dan pendidikan yang lain. Anda hanya perlu memberi angka kumpulan isi rumah anda.* | SA | Route |
| --- | --- | --- | --- |
|  |  | RM | Continue |

| Q33 | In the past 5 years, would you say about your income (Personal income)  在过去的五年里，您会认为您的收入是？  *Dalam 5 tahun yang lalu, nyatakan pendapatan anda* | SA | Route |
| --- | --- | --- | --- |
|  | Have decreased / 减少了 / Telah *berkurang* | 1 | Continue |
|  | Have remained more or less stable / 一样 /*Tidak berubah / agak stabil* | 2 |  |
|  | Have increased / 增加了 / *Telah meningkat* | 3 |  |

| Q34 | We would like to contact you for research purpose in the future.  Do you give permission to this?  您允许如果将来有需要，我们可以再联络您吗？  *Kami ingin menghubungi anda untuk tujuan penyelidikan pada masa hadapan. Adakah anda memberi kebenaran?* | SA | Route |
| --- | --- | --- | --- |
|  | Yes  愿意  Ya | 1 | END |
|  | No  不愿意  Tidak | 2 |  |

| Thank You and Close |
| --- |
